# Metformin use is associated with a low risk of tuberculosis among newly diagnosed diabetes mellitus patients with normal renal function: A nationwide cohort study with validated diagnostic criteria

**DOI:** 10.1371/journal.pone.0205807

**Published:** 2018-10-18

**Authors:** Ming-Chia Lee, Chen-Yuan Chiang, Chih-Hsin Lee, Cheng-Maw Ho, Chia-Hao Chang, Jann-Yuan Wang, Shih-Ming Chen

**Affiliations:** 1 Department of Pharmacy, New Taipei City Hospital, New Taipei City, Taiwan; 2 School of Pharmacy, College of Pharmacy, Taipei Medical University, Taipei, Taiwan; 3 Division of Pulmonary Medicine, Department of Internal Medicine, Wan Fang Hospital, Taipei Medical University, Taipei, Taiwan; 4 Division of Pulmonary Medicine, Department of Internal Medicine, School of Medicine, College of Medicine, Taipei Medical University, Taipei, Taiwan; 5 International Union Against Tubercle and Lung Disease, Paris, France; 6 Department of Surgery, National Taiwan University Hospital, Taipei, Taiwan; 7 Department of Internal Medicine, National Taiwan University Hospital, Hsinchu branch, Hsinchu, Taiwan; 8 Department of Internal Medicine, National Taiwan University Hospital, Taipei, Taiwan; Mahidol University, THAILAND

## Abstract

Human studies on the use of metformin as host-directed therapy (HDT) for tuberculosis (TB) are rare. We performed a nationwide cohort study to evaluate the effect of metformin on mitigating the risk of active TB among patients with diabetes mellitus (DM). Among newly diagnosed DM patients identified in the Taiwan National Health Insurance Research Database, metformin users, defined on the basis of >90 cumulative defined daily doses within 1 year, and propensity-score-matched metformin nonusers were selected. The primary outcome was incident TB, identified using diagnostic criteria validated by real patient data at a medical center. Independent predictors were investigated using Cox regression analysis. Similar analysis was performed in a subpopulation without a history of hypertensive nephropathy and renal replacement therapy. A total of 88,866 metformin users and 88,866 propensity-score-matched nonusers were selected. Validation results showed that the TB diagnostic criteria had a sensitivity of 99.13% and specificity of 99.90%. During follow-up, 707 metformin users and 807 nonusers developed active TB. Metformin use was independently associated with a lower risk of incident TB (hazard ratio [HR]: 0.84 [0.74–0.96]). TB risk was lower in high-dose metformin users than in low-dose users (HR: 0.83 [0.72–0.97]). The effect of metformin remained when analysis was restricted in the subpopulation without renal function impairment. Newly diagnosed diabetic patients without contraindication should receive metformin as an anti-diabetic medication, with potential additional benefit against TB.

## Introduction

Tuberculosis (TB) remains the deadliest infectious disease worldwide. In 2015, there were 10.4 million new TB cases and 1.4 million deaths due to TB [1]. Because innate and adaptive immunity are both compromised, patients with diabetes mellitus (DM) are susceptible to *Mycobacterium tuberculosis* (Mtb) infection [2], and have a threefold risk of developing active TB disease [3]. The International Diabetes Federation has estimated that 415 million adult people (8.8%) worldwide had DM in 2015, and the number of people living with diabetes will rise to 642 million by 2040 [4]. The World Health Organization has recommended a bidirectional screening for the two diseases [5].

The standard regimen for TB consists of four drugs administered for 6–9 months, which is lengthy and potentially toxic [6], especially for elderly patients [7]. The outcome of anti-TB treatment is currently suboptimal, with the treatment success rate of 73.3% in Taiwan and lower than 85% in many countries for new TB cases worldwide in 2014 [1, 8]. Host-directed therapy (HDT), which could enhance host cell response to improve pathogen eradication, is a new paradigm in drug discovery [9]. Among all potential drugs for HDT, metformin is the most commonly prescribed medication in TB patients [10, 11]. Singhal et al. reported that metformin can control the growth of drug-resistant Mtb strains in infected mice by increasing the production of mitochondrial reactive oxygen species and facilitating phagosome–lysosome fusion [10]. Metformin was associated with protection against latent TB infection (LTBI) and improvement in clinical outcome in TB patients in Singapore [10]. In an observational clinical study conducted in south India, metformin use had a protective effect against TB (odds ratio: 0.26 [0.16–0.40]) [12].

The two aforementioned human studies on the effect of metformin against TB, however, had relative small sample sizes. The Singapore study [10] enrolled only 62 DM-LTBI patients and 158 DM patients without LTBI, and the south India study [12] enrolled 451 DM cases. Furthermore, because impaired renal function is a contraindication for metformin use [13] and also a risk factor for active TB disease [14, 15], the protective effect of metformin could be confounded by impaired renal function because neither study controlled for renal function status. Moreover, the Singapore study is cross-sectional and the temporal relationship between the use of metformin and the risk of TB may not be straightforward. Therefore, we conducted a nationwide cohort study to investigate whether metformin use is associated with a lower risk of active TB disease by selecting DM patients in the National Health Insurance Research Database (NHIRD) of Taiwan.

## Methods

### Data acquisition

This cohort study used the Longitudinal Cohort of Diabetes Patients Database, a subset of the NHIRD containing the medical records from January 1, 1996, to December 31, 2013, of new DM cases (defined as DM cases without medical records for DM within 3 years before) between 2003 and 2006. For this retrospective study conducted using deidentified data, the Joint Institutional Review Board of Taipei Medical University and the Research Ethics Committee of National Taiwan University Hospital (NTUH) waived the need for informed consent (TMU-JIRB No.: N201510040; NTUH REC No.: 201702013RIN).

### Cohort selection

Patients were selected if they had at least one hospital admission or at least three outpatient visits with a DM diagnostic code (International Classification of Diseases, Ninth Revision, Clinical Modification [ICD-9-CM] code: 250) within 365 calendar days. Among such patients, those who were treated with insulin or diabetes-specific hypoglycemic agents (see S1 Text) for >90 cumulative defined daily doses (DDDs) [16] within 365 days were considered DM patients in this study. Data on individual drugs were extracted from the claims data and converted to DDDs. The agents were further grouped according to their pharmacologic categories, including insulin, sulfonylurea, meglitinide, alpha-glucosidase inhibitor, thiazolidinedione and dipeptidyl peptidase 4 (DPP-4) inhibitors. The first date of anti-DM medication usage was considered the onset of DM treatment.

DM patients were excluded if they (1) had diabetes visit claims within 270 days before parturition (to avoid including women who had gestational diabetes); (2) had a diagnosis of TB before or within the first year after the onset of DM treatment; (3) were younger than 18 years old on onset of DM treatment; or (4) had a diagnosis of end-stage renal disease or chronic kidney disease before onset of DM treatment (see S1 Text). All selected patients were followed until death, December 31, 2013, or diagnosis of TB.

### Exposure of interest: Use of metformin

Metformin users were defined as those with total prescriptions of metformin for >90 cumulative DDDs within 1 year after the onset of DM treatment. For each metformin-using DM patient, a propensity-score (PS)-matched metformin nonuser was selected. Metformin users were further stratified into two subgroups (high-dose users *vs*. low-dose users) according to a cumulative dose cutoff of 150 DDDs. The cumulative DDDs of metformin within each subsequent year were also calculated. The index date in this study was defined as one year after the onset of DM treatment.

### Outcome of interest: Incident TB

The outcome of interest was newly diagnosed TB after the index date. The diagnosis of TB was considered definite for patients who had at least two ambulatory visits or one inpatient record with a compatible diagnosis (ICD-9-CM code: 010–018), plus at least one prescription of three or more anti-TB drugs and prescription of at least two anti-TB drugs simultaneously for 120 days within a period of 180 days (see S1 Text for drug list). The diagnosis of TB was considered probable for patients who had a Mtb drug susceptibility test or used at least two anti-TB drugs for more than 1 month with at least one prescription of three or more anti-TB drugs within the final 3 months before death or loss to follow-up. Those who were diagnosed with non-TB mycobacteria (ICD-9-CM code: 031) before 60 days of the date of completion of anti-TB treatment were not considered to be TB cases [17]. Both definite and probable TB cases were considered outcome events.

### Validation for the diagnostic criteria of TB

The aforementioned diagnostic criteria of TB were validated by examining patients suspected to have TB who had sputum acid-fast smear and mycobacterial cultures between January 2011 and December 2012 at NTUH, a medical center in northern Taiwan. TB was confirmed if (1) clinical specimens were culture-positive for Mtb; (2) biopsy specimens had granulomatous inflammation or acid-fast bacilli, and the clinical condition improved after anti-TB treatment; or (3) typical symptoms of TB were alleviated after anti-TB treatment. TB patients who were transferred to other hospitals during treatment were excluded.

### Possible confounding factors

In the Longitudinal Cohort of Diabetes Patients Database, underlying comorbidities that can potentially influence the risk of TB were recorded at the index date. The comorbidities, defined according to previous studies, are provided in the S1 Text, including chronic obstructive pulmonary disease (COPD), pulmonary cancer, extra-pulmonary cancer, liver cirrhosis, acquired immunodeficiency disease, pneumoconiosis, bronchiectasis, rheumatoid arthritis, ankylosing spondylitis, psoriasis, and severe autoimmune diseases [17, 18].

Patients were considered to have type 1 DM if any two of the following criteria were fulfilled: (1) outpatient or inpatient diagnoses of type 1 DM (ICD-9-CM code: 250.x1 or 250.x3); (2) admission for diabetic ketoacidosis (ICD-9-CM code 250.1); or (3) compatible ICD-9-CM codes in the Registry for Catastrophic Illness Patient Database. DM chronic complications were DM nephropathy (ICD-9-CM code: 250.4), DM retinopathy (ICD-9-CM code: 250.5), DM neuropathy (ICD-9-CM code: 250.6), DM vasculopathy (ICD-9-CM code: 250.7), and other DM complications (ICD-9-CM code: 250.8, 250.9).

Use of systemic immunosuppressants & biological agents, disease-modifying antirheumatic drugs (DMARDs), corticosteroids, statin, oral hypoglycemic agents (OHAs) other than metformin, aspirin, non-steroidal anti-inflammatory drugs (NSAIDs) and calcium channel blockers (CCBs) (see S1 Text for drug list) was identified on the basis of prescriptions in each category for >90 cumulative DDDs within 1 year before the index date (between onset of DM treatment and index date). Use of insulin was defined as more than one prescription of insulin at the outpatient clinic within 1 year before the index date. The low-income group was defined as an annual household income lower than 4,500 US dollars [17, 18].

### Statistical analysis

To increase the comparability between the metformin users and nonusers, PS matching was performed using a multivariate logistic regression model to estimate and control the probability of receiving metformin. The variables included age at the onset date of DM, sex, type 1 DM, income, chronic obstructive pulmonary disease (COPD), pulmonary cancer, extra-pulmonary cancer, liver cirrhosis, acquired immunodeficiency disease, pneumoconiosis, bronchiectasis, rheumatoid arthritis, ankylosing spondylitis, psoriasis, and severe autoimmune diseases, DM chronic complications, and use of systemic immunosuppressants & biological agents, DMARDs, corticosteroids, statin, insulin, OHAs other than metformin, aspirin, NSAIDs and CCBs (all listed in Table 1).

**Table 1:** Clinical characteristics of metformin users stratified by dose and propensity score-matched nonusers.

Characteristics	Metformin nonusers	Metformin users				*p*-value^#^
	All (n = 88,866)	All (n = 88,866)	Lose-dose (n = 36,756)	High-dose (n = 52,110)	*p*-value*	
Male	48270 (54.3%)	48,345 (54.4%)	19,891 (54.1%)	28,454 (54.6%)	0.151	0.724
Age (mean ± SD)	55.9±13.1	55.9±12.9	56.3±13.0	55.7±12.8	<0.001	0.697
Type 1 DM	1,920 (2.2%)	1905 (2.1%)	742 (2.0%)	1163 (2.2%)	0.031	0.819
Co-morbidity						
COPD	4,821 (5.4%)	4,790 (5.4%)	2,116 (5.8%)	2,674 (5.1%)	<0.001	0.748
Pulmonary cancer	129 (0.1%)	119 (0.1%)	47 (0.1%)	72 (0.1%)	0.679	0.568
Extra-pulmonary cancer	2,453 (2.8%)	2434 (2.7%)	997 (2.7%)	1437 (2.8%)	0.685	0.794
Bronchiectasis	724 (0.8%)	733 (0.8%)	302 (0.8%)	431 (0.8%)	0.929	0.832
Psoriasis	598 (0.7%)	595 (0.7%)	233 (0.6%)	362 (0.7%)	0.274	0.954
Rheumatoid arthritis	330 (0.4%)	320 (0.4%)	165 (0.4%)	155 (0.3%)	<0.001	0.724
Ankylosing spondylitis	190 (0.2%)	185 (0.2%)	84 (0.2%)	101 (0.2%)	0.264	0.836
Liver cirrhosis	164 (0.2%)	162 (0.2%)	69 (0.2%)	93 (0.2%)	0.750	0.955
Severe autoimmune disease	134 (0.2%)	128 (0.1%)	56 (0.2%)	72 (0.1%)	0.583	0.757
Pneumoconiosis	106 (0.1%)	95 (0.1%)	40 (0.1%)	55 (0.1%)	0.883	0.481
HIV/AIDS	48 (0.05%)	40 (0.04%)	19 (0.05%)	21 (0.04%)	0.43	0.456
Transplantation	43 (0.04%)	40 (0.04%)	13 (0.04%)	27 (0.05%)	0.255	0.822
DM chronic complication^$^	5,909 (6.6%)	6,039 (6.8%)	2289 (6.2%)	3750 (7.2%)	<0.001	0.218
Low income	6,172 (6.9%)	6,135 (6.9%)	2560 (7.0%)	3575 (6.9%)	0.546	0.737
Medication use						
Insulin	4,632 (5.2%)	4,743 (5.3%)	2,005 (5.5%)	2,738 (5.3%)	0.190	0.243
OHAs other than metformin^&^	59,747 (67.2%)	58,820 (66.2%)	22,538 (61.3%)	36,282 (69.6%)	<0.001	<0.001
Statin	16,879 (19.0%)	17,644 (19.9%)	6,355 (17.3%)	11,289 (21.7%)	<0.001	<0.001
Aspirin	17,494 (19.7%)	17,527 (19.7%)	6,723 (18.3%)	10,804 (20.7%)	<0.001	0.837
CCBs	26,463 (29.8%)	26,246 (29.5%)	10,324 (28.1%)	15,922 (30.6%)	<0.001	0.245
NSAIDs	6,762 (7.6%)	6,768 (7.6%)	2,880 (7.8%)	3,888 (7.5%)	0.038	0.964
Corticosteroids	4,491 (5.1%)	4,575 (5.1%)	2,012 (5.5%)	2,563 (4.9%)	<0.001	0.366
Immunosuppressants & biologicals	8 (0.01%)	8 (0.01%)	2 (0.01%)	6 (0.01%)	0.347	>0.999
DMARDs	4 (0.004%)	2 (0.002%)	1 (0.003%)	1 (0.002%)	0.804	0.688

Abbreviations: AIDS, acquired immunodeficiency syndrome; CCBs, calcium channel blockers; COPD, chronic obstructive pulmonary disease; DM, diabetes mellitus; DMARD, disease modifying antirheumatic drug; NSAIDs, non-steroidal anti-inflammatory drugs; OHAs, oral hypoglycemic agents.

Data are expressed as the number (%) unless otherwise specified.

* *p* value of low-dose *vs*. high-dose metformin users in independent-samples *t* test for continuous variables and *chi*-square test for categorical variables.

^#^ *p* value of metformin users *vs*. nonusers in paired *t* test for continuous variables and McNemar test for categorical variables.

^$^ Including diabetic nephropathy, diabetic retinopathy, diabetic neuropathy, and diabetic vasculopathy.

^&^ Including sulfonylurea, meglitinide, alpha-glucosidase inhibitor, thiazolidinedione, dipeptidyl peptidase-4 (DDP4)-inhibitor.

Data were expressed as the mean ± standard deviation or number (%), as appropriate. Intergroup differences for categorical and continuous variables were evaluated using the McNemar test and paired *t* test for matched samples and the *chi*-squared test and independent-samples *t* test for unrelated samples. Kaplan–Meier curves for time to incident TB among the metformin users and nonusers were generated and compared using the log-rank test. Multivariate stratified Cox proportional hazard regression analysis was used to identify the independent factors associated with incident TB for all DM patients by adjusting sex, type 1 diabetes mellitus, age, low income, chronic obstructive pulmonary disease, liver cirrhosis, pulmonary cancer, Extra-pulmonary cancer, bronchiectasis, psoriasis, rheumatoid arthritis, ankylosing spondylitis, severe autoimmune disease, pneumoconiosis, acquired immunodeficiency syndrome, transplantation, chronic complications of diabetes mellitus, use of insulin, OHAs other than metformin, statin, aspirin, CCBs, NSAIDs, steroid, immunosuppressants & biologicals, and DMARDs, as well as interaction between metformin and statin, metformin and aspirin, metformin and NSAIDs, and metformin and CCBs. In dose–response analysis, only the metformin users were included in the Cox regression by adjusting the same variables. All analyses were performed using SPSS (IBM Corp. IBM SPSS Statistics for Windows, Version 24.0. Armonk, NY: IBM Corp.)

### Subpopulation analysis

Because impaired renal function was a contraindication for using metformin [13] and was also a risk factor for developing TB [14, 15], a subpopulation analysis was performed in patients having no previous diagnosis of diabetic nephropathy, hypertensive nephropathy or history of renal replacement therapy (see S1 Text for details) before the index date. Sub-group analysis for sex, the use of insulin, OHAs other than metformin, and statin were also performed.

## Results

### Case selection and clinical characteristics

**Fig 1:**
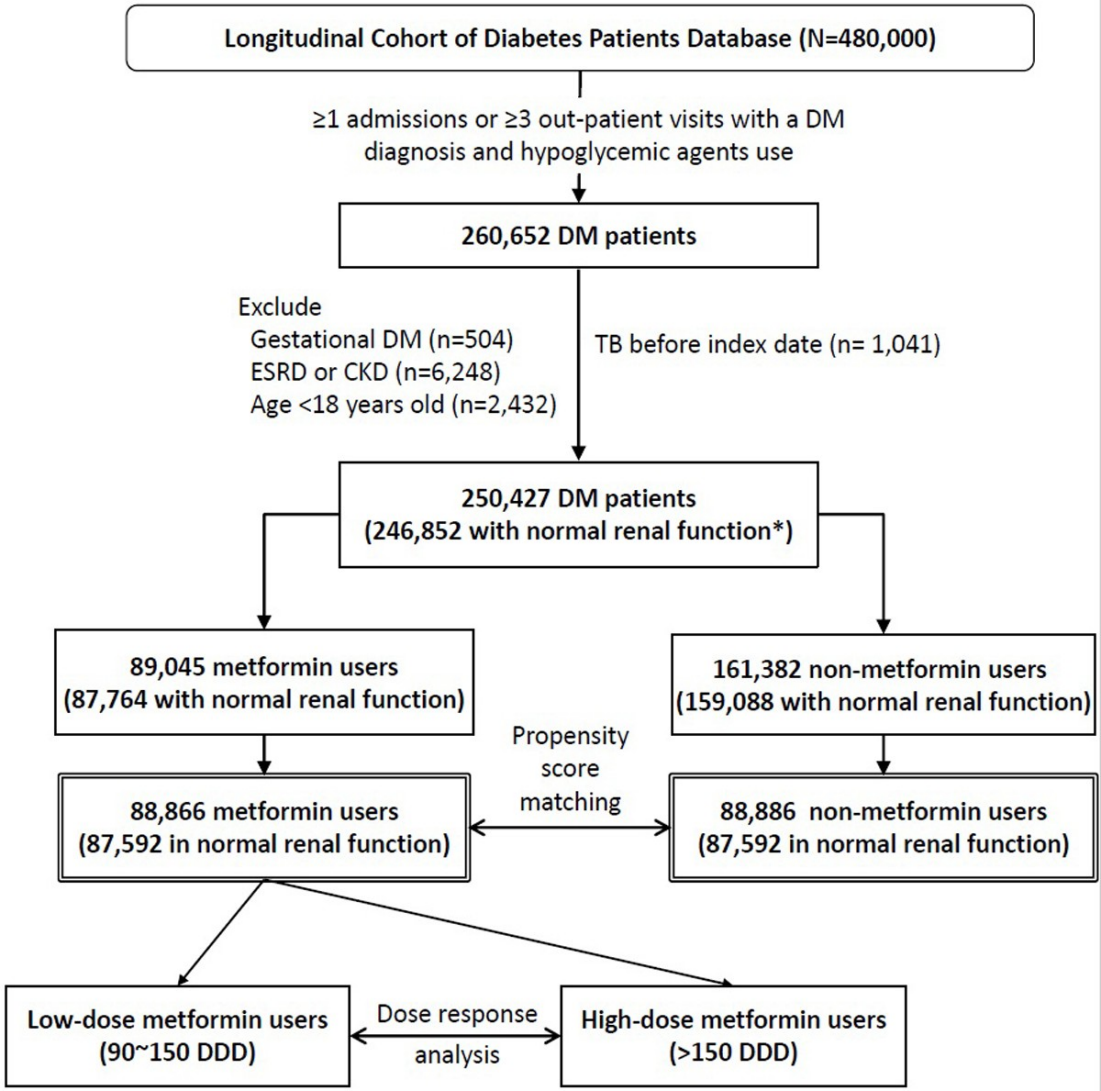
Flowchart of study design and case selection (CKD: Chronic kidney disease; DM: Diabetes mellitus; ESRD: End-stage renal disease; TB: Tuberculosis; * normal renal function was defined as no previous diagnosis of hypertensive nephropathy and no receipt of renal replacement therapy before the index date).

Among the 480,000 DM patients in the Longitudinal Cohort of Diabetes Patients Database, 250,427 had newly diagnosed DM (Fig 1). Of them, 89,045 were classified as metformin users. After PS matching, 88,866 metformin users and 88,866 metformin nonusers were selected for this study. The baseline characteristics of 179 unmatched metformin users are described in the S1 Table.

The baseline characteristics were balanced between the metformin users and nonusers (Table 1). The mean age was 55.9 years. Male predominance was noted in both groups. The most common underlying comorbidities were chronic obstructive pulmonary disease (COPD) (5.4%) and malignancy (2.9%). The proportions of patients with type 1 DM, DM chronic complications, and low income were 2.2%, 6.5%, and 6.9%, respectively. Only 0.05% had HIV/AIDS. Among the concomitant medications, OHAs other than metformin (67.2%) and CCBs (29.8%) were the most commonly used. The metformin users received higher cumulative DDDs of metformin within each subsequent year (Figure A in S1 File).

In comparison with the low-dose metformin users, the high-dose metformin users were younger (55.7 ± 12.8 years *vs*. 56.3 ± 13.0 years), more likely to have type 1 DM and DM chronic complications (7.2% *vs*. 6.2%), more likely to receive statin (21.7% *vs*. 17.3%), aspirin (20.7% *vs*. 18.3%), and CCBs (30.6% *vs*. 28.1%), less likely to have COPD (5.1% *vs*. 5.8%) and rheumatoid arthritis (0.3% *vs*. 0.4%), and less likely to use corticosteroids (4.9% *vs*. 5.5%).

### Validation for the diagnostic criteria of TB

From January 2011 to December 2012, 20,720 patients suspected to have TB were identified at NTUH (S2 Table). Among them, TB was culture-confirmed in 633, histologically proven in 103, and clinically diagnosed in another 69. Of these 805 TB patients, the NHIRD TB diagnostic criteria identified all except six patients who experienced multiple adverse events requiring treatment interruption in several occasions, and another patient with histologically proven TB peritonitis died within 1 month after treatment. Of the 19,915 non-TB patients, two were identified as definite TB cases by the NHIRD diagnostic criteria. One had bladder infection after bladder instillation of bacillus Calmette–Guérin for transitional cell carcinoma. The other patient was culture-negative for Mtb in all clinical samples and the diagnosis of TB was excluded after 4.5 months of treatment. Furthermore, there were 18 patients who died soon after empiric anti-TB treatment and were identified as probable TB cases. Their clinical samples were all culture-negative for Mtb. The sensitivity and specificity of the NHIRD TB diagnostic criteria were 99.13% and 99.90%, respectively. The positive and negative predictive values were 97.56% and 99.96%, respectively.

### Incident TB cases in the NHIRD

During follow-up, 707 metformin users and 807 metformin nonusers developed active TB disease, corresponding to 127 and 140 cases per 100,000 person-years, respectively (*p* = 0.012). Within 2, 4, and 6 years after the index date, the cumulative number of incident TB cases was 351 (49.6%), 541 (76.5%), and 665 (94.1%) among the metformin users, respectively, and 418 (51.8%), 613 (76.0%), and 769 (95.3%) among the metformin nonusers, respectively.

**Fig 2:**
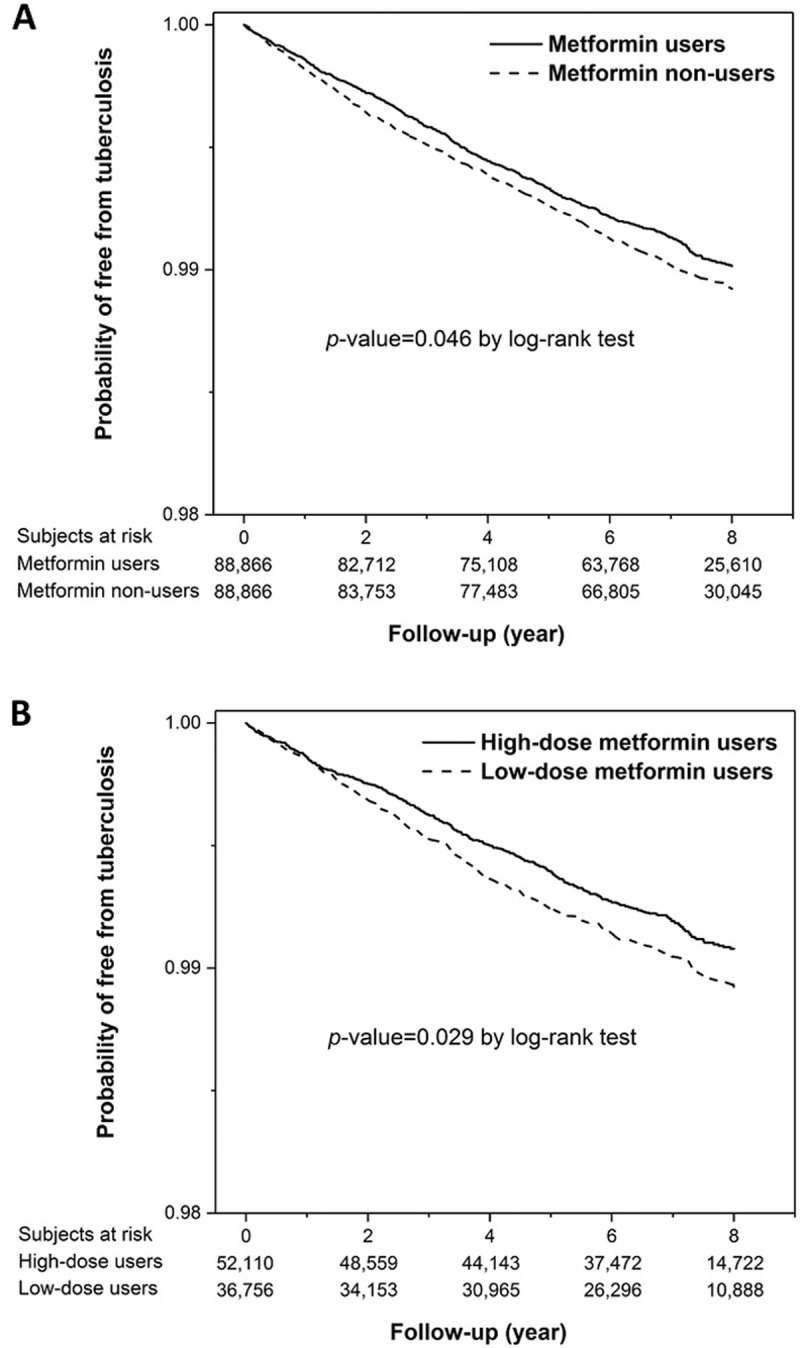
Kaplan–Meier curves depicting time to active tuberculosis among metformin users and nonusers (2A), and high-dose and low-dose metformin users (2B).

Time to TB significantly differed between the metformin users and nonusers (*p* = 0.046 by log-rank test) (Fig 2A) and between the high-dose and low-dose metformin users (*p* = 0.029 by log-rank test) (Fig 2B).

### Risk factors for TB in newly-diagnosed DM patients

Multivariate Cox proportional hazard regression analysis revealed that metformin use (>90 cumulative DDDs within one year, i.e. between onset of DM treatment and index date) was independently associated with a lower risk of incident TB (*p* = 0.013; hazard ratio [HR]: 0.84 [0.74–0.96]) (Table 2). Use of statin (HR: 0.29 [0.17–0.48]), aspirin (HR: 0.68 [0.50–0.91]) and CCBs (HR: 0.65 [0.51–0.82]) were also protective. However, concurrent use of metformin and statin was a risk factor for developing TB (HR: 1.60 [1.08–2.37]), implying a negative interaction. The male sex (HR: 2.90 [2.39–3.52]), and type 1 DM (HR: 3.68 [2.03–6.68]) were the most potent risk factors for developing TB. Other independent risk factors included age (HR: 1.046 [1.034–1.058] per year increment), COPD (HR: 1.82 [1.27–2.61]), insulin use (HR: 2.03 [1.43–2.89]), NSAIDs use (HR: 1.38 [1.03–1.86]) and steroid use (HR: 1.75 [1.47–2.07]).

**Table 2:** Predictors of tuberculosis development among newly diagnosis diabetic patients determined through multivariate stratified Cox proportional hazard regression analysis after propensity score matching for metformin use.

Variables	*p*-value	Hazard ratio (95% CI)
Metformin user	0.013	0.84 (0.74–0.96)
Statin user	<0.001	0.29 (0.18–0.48)
Statin x metformin user	0.021	1.60 (1.08–2.37)
Aspirin user	0.011	0.68 (0.50–0.91)
CCB user	<0.001	0.65 (0.51–0.82)
Male sex	<0.001	2.90 (2.39–3.52)
Type 1 diabetes mellitus	<0.001	3.68 (2.03–6.68)
Age (per year increment)	<0.001	1.046 (1.034–1.058)
Chronic obstructive pulmonary disease	<0.001	1.82 (1.27–2.61)
Steroid user	0.003	1.73 (1.21–2.47)
Insulin user	<0.001	2.03 (1.43–2.89)
NSAID user	0.033	1.38 (1.03–1.86)

Abbreviations: CCB, calcium channel blocker; NSAID, non-steroidal anti-inflammatory drug;

Adjusted variables included male, type 1 diabetes mellitus, age, low income, chronic obstructive pulmonary disease, liver cirrhosis, pulmonary cancer, extra-pulmonary cancer, bronchiectasis, psoriasis, rheumatoid arthritis, ankylosing spondylitis, severe autoimmune disease, pneumoconiosis, acquired immunodeficiency syndrome, transplantation, chronic complications of diabetes mellitus, insulin user, use of OHAs other than metformin, statin user, aspirin user, CCB user, NSAID user, steroid user, immunosuppressants & biologicals user, DMARDs user and interaction between metformin and statin, metformin and aspirin, metformin and NSAIDs, and metformin and CCBs.

### Dose–response analysis for metformin users

The baseline characteristics of the high- and low-dose metformin users in the dose–response analysis are shown in S4 Table. Multivariate analysis revealed that cumulative metformin use >150 DDDs was associated with a significantly lower risk of incident TB after the index date than cumulative metformin use between 90 and 150 DDDs (HR: 0.83 [0.72–0.97]) (Table 3). Use of CCBs was still protective (HR 0.77 [0.65–0.91]), however, use of statin and aspirin was not protective in the metformin users. The most potent risk factors were the male sex (HR 2.99 [2.51–3.58]) and insulin user (HR: 1.92 [1.51–2.45]). Other independent risk factors included age (HR: 1.031 [1.025–1.037]), type 1 DM (HR: 1.66 [1.13–2.43]), COPD (HR: 1.44 [1.12–1.86]), the use of OHAs other than metformin (HR: 1.83 [1.52–2.20]) and steroid (HR: 1.75 [1.36–2.26]).

**Table 3:** Dose–response analysis for the effect of metformin use on tuberculosis development in the 88,866 diabetic metformin users (receiving ≥90 defined daily doses [DDDs] of metformin within the first year after the index date).

Variables	*p*-value	Hazard ratio (95% CI)
Metformin dose: >150 *vs*. 90–150 DDDs	0.015	0.83 (0.72–0.97)
CCB user	0.003	0.77 (0.65–0.91)
Male sex	<0.001	2.99 (2.51–3.58)
Type 1 diabetes mellitus	<0.001	1.66 (1.13–2.43)
Age (per year increment)	<0.001	1.031 (1.025–1.037)
Chronic obstructive pulmonary disease	0.005	1.44 (1.12–1.86)
Steroid user	<0.001	1.75 (1.36–2.26)
Insulin user	<0.001	1.92 (1.51–2.45)
Use of OHAs other than metformin	<0.001	1.83 (1.52–2.20)

Abbreviations: CCB, calcium channel blocker; OHAs, oral hypoglycemic agents

Adjusted variables included male, type 1 diabetes mellitus, age, low income, chronic obstructive pulmonary disease, liver cirrhosis, pulmonary cancer, extra-pulmonary cancer, bronchiectasis, psoriasis, rheumatoid arthritis, ankylosing spondylitis, severe autoimmune disease, pneumoconiosis, acquired immunodeficiency syndrome, transplantation, chronic complications of diabetes mellitus, insulin user, use of OHAs other than metformin, statin user, aspirin user, CCB user, non-steroidal anti-inflammatory drugs (NSAIDs) user, steroid user, immunosuppressants & biologicals user, disease-modifying antirheumatic drugs (DMARDs) user and interaction between metformin and statin, metformin and aspirin, metformin and NSAIDs, and metformin and CCBs.

### Subpopulation analysis

Among the 88,866 metformin users, 87,592 had normal renal function and, together with 87,592 PS-matched metformin nonusers with normal renal function, were selected for subpopulation analysis. The clinical characteristics were balanced between the two subgroups (S3 Table). Among diabetic patients with normal renal function, metformin users had higher metformin cumulative DDDs within each subsequent year after the index date than metformin nonusers (Figure B in S1 File).

Multivariate stratified Cox regression analysis revealed that metformin use was independently associated with a lower risk of active TB disease (HR: 0.82 [0.72–0.95]) among diabetic patients with normal renal function, similar to that in the total study population (Table 4). The high-dose metformin users (>150 cumulative DDDs) had a significantly lower risk of incident TB than the low-dose metformin users (90–150 cumulative DDDs) (HR: 0.84 [0.73–0.98]).

**Table 4:** Impact of metformin use on tuberculosis development in the whole study population and patients with normal renal function.

	*p*-value	Hazard ratio (95% CI)
All subjects (n = 177,732)	0.013	0.84 (0.74–0.96)
Subgroup with normal renal function* (n = 175,184)	0.006	0.82 (0.72–0.95)
Male (n = 48,345)	0.078	0.90 (0.80–1.01)
Female (n = 40,521)	0.076	0.83 (0.67–1.02)
Insulin user (n = 4,743)	0.489	1.12 (0.81–1.55)
Insulin nonuser (n = 84,123)	0.011	0.87 (0.78–0.97)
Using OHAs other than metformin (n = 58,852)	0.33	1.06 (0.94–1.20)
Not using OHAs except metformin (n = 30,046)	<0.001	0.57 (0.46–0.71)
Not using DM medication except metformin (n = 29,281)	<0.001	0.51 (0.39–0.66)
Statin user (n = 17,644)	0.232	1.18 (0.90–1.54)
Statin nonuser (n = 71,222)	0.006	0.86 (0.77–0.96)

Abbreviations: DM, diabetes mellitus; OHAs, oral hypoglycemic agents;

Cox proportional regression was adjusted by variables including male, type 1 diabetes mellitus, age, low income, chronic obstructive pulmonary disease, liver cirrhosis, pulmonary cancer, extra-pulmonary cancer, bronchiectasis, psoriasis, rheumatoid arthritis, ankylosing spondylitis, severe autoimmune disease, pneumoconiosis, acquired immunodeficiency syndrome, transplantation, chronic complications of diabetes mellitus, insulin user, use of OHAs other than metformin, statin user, aspirin user, calcium channel blocker (CCB) user, non-steroidal anti-inflammatory drugs (NSAIDs) user, steroid user, immunosuppressants & biologicals user, drug-modifying antirheumatic drugs (DMARDs) user, and interaction between metformin and statin, metformin and aspirin, metformin and NSAIDs, and metformin and CCBs.

* Defined as no previous diagnosis of hypertensive nephropathy and no receipt of renal replacement therapy before the index date.

Subpopulation analysis revealed that metformin was protective from developing TB diseases in many subgroups, including patients not using insulin (HR: 0.87 [0.78–0.97]), patients not using OHAs other than metformin (HR: 0.57 [0.46–0.71]), and patients using metformin but not insulin nor other OHAs (HR: 0.51 [0.39–0.66]), and patients not using statin (HR: 0.86 [0.77–0.96]).

## Discussion

By using a nationwide DM cohort with outcomes validated by real patient data at a medical center and controlling for the confounding effect of renal function, this study demonstrates an independent effect of metformin in protecting DM patients from active TB disease. The study has two major findings. First, DM patients receiving metformin for >90 cumulative DDDs within 1 year have a lower risk of incident TB. Second, the protective effect of metformin use is dose dependent, with a cumulative dose of >150 DDDs within 1 year being associated with a lower TB risk compared with 90–150 cumulative DDDs.

Active TB disease, identified according to validated and precise diagnostic criteria, was used as the primary endpoint in this study. Patients with LTBI have a lifetime risk of progression to active TB of 10%, with half of them experiencing progression within the first 2–5 years [19]. Therefore, the impact of metformin use on TB prevention can be properly measured through longitudinal follow-up of a large DM cohort. By using a nationwide database, the current study demonstrates that metformin protects against active TB disease in a dose response manner. DM patients who received more than 150 DDDs of metformin within 1 year are less likely to have incident TB than those receiving 90–150 DDDs. According to the Bradford Hill criteria, the biological gradient (dose–response) is one of the important criteria confirming a causal relationship [20]. Based on the results of this study indicating that the TB incidence was approximately 140 per 100,000 patient-years in DM patients not using metformin and that metformin use could provide a 15%–16% reduction in the risk of TB disease in DM patients, we estimate that universal use of metformin would prevent 78,435–91,507 TB cases in the overall 415-million-person adult DM population worldwide.

In assessing the independent protective effect of metformin against active TB disease in DM patients, it is critical to control for multiple comorbidities (such as COPD, liver cirrhosis, and steroid use) [21], which tend to coexist and contribute to outcome development, as demonstrated in the current study. DM may be one of the manifestations of metabolic syndrome; diabetic patients have a high prevalence of hyperlipidemia [22] and are likely to receive statin—another candidate for HDT against active TB disease [23]. In addition, the prevalence of DM increases with age, which is also an independent risk factor of active TB disease though its impact maybe trivial. Furthermore, other prevalent comorbidities in the aging population, such as COPD [24], may increase the risk of active TB disease [25].

Neither of the two previous human studies demonstrating the association between metformin use and a lower TB risk [10, 12] controlled for the potential confounding effect of poor renal function. Metformin is contraindicated in patients with renal function impairment defined as an estimated glomerular filtration rate < 30 mL/min/1.73m^2^ [13]. Compared with the general population, patients with chronic renal failure and on dialysis have a 3.62-fold increased risk of TB [15]. Even in those with acute kidney injury requiring dialysis, the HR for active TB was 7.71 relative to the general population [26]. Therefore, the benefits of metformin use in the two studies may be confounded by indication, rather than represent a true protective effect.

Pan et al. recently conducted a cohort study of DM patients using the Longitudinal Health Insurance Database of Taiwan and excluded those with chronic kidney disease. They indicated a similar result that use of metformin could provide 66% reduction of TB risk compared to use of sulfonylurea (HR 0.34 [0.17–0.67]) [27]. However, they did not discuss the use of insulin, OHAs other than metformin, and other HDT drugs, such as aspirin, NSAIDs and CCBs. In addition, the interaction between metformin and other HDT drugs was not addressed.

Statin have been shown to reduce cholesterol levels within phagosomal membranes and counteract the Mtb-induced inhibition of phagosomal maturation to promote host-induced autophagy in human macrophages and experimental mouse models [28]. By controlling multiple risk factors of TB and several HDT drugs as well as their interactions in a Cox model, the results of the current study confirmed the independent protective effect of metformin against active TB. In line with previous studies [29–31], it also demonstrates that statin (HR 0.29 [0.18–0.48]), aspirin (HR 0.68 [0.50–0.91]) and CCBs (HR 0.65 [0.51–0.82]) may reduce the risk of active TB. The finding that metformin and statin has a negative interaction in TB protection is surprising, but may partially explain the discrepant findings in different studies on metformin as a HDT for TB [10, 32–34]. Though the current study is not designed to explore the pathophysiology underlying this phenomenon, we speculate that this may probably because metformin and statin both target on facilitation of phagosome–lysosome fusion [10], and therefore counteract each other. However, a perspective clinical study to confirm the protective effect and the potential interaction between these HDT drugs is needed.

It is interesting that the protective effect of metformin wanes in DM patients receiving either insulin or other OHAs. The therapeutic option for DM patients who fail to control in blood sugar well by lifestyle intervention and metformin is to add a second OHAs or insulin, or both [35]. Those patients may have a poor-controlled blood sugar, which could increase the risk of active TB diseases [36] and counteract the protective effects of metformin use. In our study, NSAIDs increased the risk of TB, which is a similar finding in a previous study [37]. Whether the association between traditional NSAIDs and TB is causal, or simply reflects an increased use of anti-inflammatory drugs in the early phases of TB onset remains to debate [37]. A systemic review, however, indicated an opposite result, showing a beneficial effect of NSAIDs as an adjunct to current TB therapy regimens [38]. Further studies are needed to confirm these findings.

This study has some limitations. First, since data on fasting blood sugar and hemoglobin A1c are lacking in the NHIRD, we were not able to identify DM patients with laboratory data and assess the quality of glycemic control. Instead, DM cohort was selected by compatible diagnosis code and confirmed by prescription of anti-diabetic medications. Therefore, DM patients who did not receive any anti-diabetic medications were not included in the study. In addition, it is difficult to distinguish the protective effect of metformin on TB from good glycemic control though we have used the presence of diabetic chronic complications as a surrogate of glycemic control [39, 40]. Second, the body mass index (BMI) is another important consideration in selecting hypoglycemic agents [41]. Low body weight (BMI < 18.5) has been shown to increase the risk of TB by a factor of 2.6 (1.2–4.8) [42, 43]. However, information on BMI is lacking in the NHIRD. Third, we used only the cumulative DDDs of metformin within one year before the index date to classify metformin users and nonusers. However, this may not be a severe problem because metformin users received higher cumulative DDDs of metformin within each subsequent year and the Kaplan–Meier curves for metformin users and nonusers continue to run separately for 8 years, indicating a possible positive correlation with short-term metformin exposure and long-term immunity against TB infection [12]. Lastly, because metformin is the first-line therapy for patients with type 2 DM if without any contraindications [44], metformin nonusers might still receive metformin in a lower dose (≤90 cumulative DDDs per year) and thus have some benefit from metformin in preventive active TB disease. If this is the case, the rate of TB among patients receiving no metformin should be higher than that in the metformin nonusers in the study, thus biasing the results towards the null. As our results, a better compliance to metformin (>90 cumulative DDDs) protects from active TB disease.

## Conclusion

The findings of this nationwide cohort study suggest that the use of metformin in DM patients reduces the risk of subsequent active TB, and the protection is dose responsive. Healthcare professionals should consider prescribing metformin for primary prevention of active TB in DM patients if no contraindications exist.

## Supporting information

S1 Text Definitions of comorbidities, drug code and management code. (DOCX)

S1 Table Clinical characteristics of matched and unmatched metformin users. (DOCX)

S2 Table Results of validation for the diagnostic criteria of tuberculosis (TB). (DOCX)

S3 Table Clinical characteristics of metformin users and nonusers among diabetic patients with normal renal function after propensity score matching. (DOCX)

S4 Table Clinical characteristics of metformin users with normal renal function stratified by cumulative metformin dose using a cutoff of 150 defined daily doses (cDDDs). (DOCX)

S1 File Cumulative defined daily dose (DDD) of metformin in metformin users and nonusers in each year among all enrolled patients with diabetes mellitus (Figure A) and those with normal renal function (Figure B).
(DOCX)

S1 Dataset Original dataset for analysis. (CSV)
