# Supplementary material for: Metformin use is associated with a low risk of tuberculosis among newly diagnosed diabetes mellitus patients with normal renal function: A nationwide cohort study with validated diagnostic criteria
Source: PLoS One. 2018 Oct 18;13(10):e0205807. doi: 10.1371/journal.pone.0205807 (PMC6193668; doi:10.1371/journal.pone.0205807)
Supplement: S1 Text — (DOCX) [file pone.0205807.s001.docx]

**Definitions of Comorbidities**

**Chronic obstructive pulmonary disease (COPD)**: at least two outpatient or inpatient records with compatible diagnoses (ICD-9-CM codes 491, 492, 496) and a prescription of at least two COPD-specific medications or one COPD-specific medication plus at least one airway medication in 90 days. (The COPD-specific medications included corticosteroids [inhaled, oral, or parenteral], β-agonists [long-acting or short-acting; inhaled or oral], anticholinergics [ipratropium or tiotropium], aminophylline, and theophylline. Airway medications included oral antitussives, mucolytic agents, and sympathomimetics.)

**Pulmonary cancer**: compatible ICD-9-CM code (162) from the Registry for Catastrophic Illness Patient Database (RCIPD), which is a separate file section of the National Health Insurance Database and requires histologic, radiologic, or laboratory confirmation of the disease.

**Extra-pulmonary cancer:** ICD-9-CM codes 140–208, but not 162, from the RCIPD.

**Liver cirrhosis:** ICD-9-CM code 571 in the RCIPD.

**Acquired immunodeficiency disease**: two or more records with compatible diagnoses (ICD-9-CM codes 042, V08) and prescription of highly active antiretroviral therapy in 180 days.

**Pneumoconiosis**: ICD-9-CM codes 500–505 in the RCIPD.

**Bronchiectasis**: At least two outpatient records or one inpatient record of ICD-9-CM code 494 within 90 days.

**Rheumatoid arthritis**: At least three outpatient records of ICD-9-CM code 714 entered by a rheumatologist or dermatologist within 180 days or one inpatient record of ICD-9-CM code 714.

**Ankylosing spondylitis**: At least three outpatient records of ICD-9-CM code 720 entered by a rheumatologist or dermatologist within 180 days or one inpatient record of ICD-9-CM code 720.

**Psoriasis**: At least three outpatient records of ICD-9-CM code 696 entered by a rheumatologist or dermatologist within 180 days or one inpatient record of ICD-9-CM code 696.

**Severe autoimmune diseases:** ICD-9-CM code 710 or 714 in the RCIPD.

**End-stage renal disease and chronic kidney disease:** compatible diagnoses (ICD-9-CM code 585) in at least two outpatient records, at least one inpatient record, or in the RCIPD.

**Hypertensive nephropathy:** ICD-9-CM code 403.

**List of Drugs and Anatomical Therapeutic Chemical (ATC) Codes**

**Antituberculosis drugs:** isoniazid, rifampin, rifabutin, ethambutol, pyrazinamide, prothionamide, terizidone, streptomycin, kanamycin, quinolones, cycloserine, and aminosalicylic acid (ATC codes: J04AA, J04AB, J04AC, J04AD, J04AK, J04AM)

**Aspirin:** ATC code: B01AC06, N02BA01

**Calcium channel blocker:** ATC code: C08CA

**Corticosteroids:** ATC code: H02AB, H02B

**Diabetes-specific hypoglycemic agents:** acarbose, acetohexamide, buformin, chlorpropamide, glibornuride, gliclazide, glimepiride, glipizide, gliquidone, glyburide, insulin, metformin, nateglinide, pioglitazone, repaglinide, rosiglitazone, tolazamide, and tolbutamide (ATC codes: A10BA, A10BB, A10BD, A10BF, A10BG, A10BH, A10BX)

**Disease-modifying antirheumatic drugs (DMARDs):** quinolines, gold preparations, penicillamine, and other similar agents (ATC codes: M01CA, M01CB, M01CC)

**Non-steroid anti-inflammatory drug (NSAID):** ATC code: M01AA, M01AB, M01AC, M01AE, M01AG, M01AH, M01AX

**Statins:** ATC code: C10AA, C10BA, C10BX

**Systemic immunosuppressants and biological agents:** selective immunosuppressants, tumor necrosis factor alpha inhibitors, interleukin inhibitors, calcineurin inhibitors, and other immunosuppressants (ATC codes: L04AA, L04AB, L04AC, L04AD, and L04AX)

**Management Codes**

**Renal replacement therapy:** including hemodialysis (58001C, 58026C, 58027C, 58029C, 58030B) and peritoneal dialysis (58002C, 58009B, 58010B, 58011C, 58012B, 58013C, 58017C, 58028C)
