# Supplementary material for: Metformin use is associated with a low risk of tuberculosis among newly diagnosed diabetes mellitus patients with normal renal function: A nationwide cohort study with validated diagnostic criteria
Source: PLoS One. 2018 Oct 18;13(10):e0205807. doi: 10.1371/journal.pone.0205807 (PMC6193668; doi:10.1371/journal.pone.0205807)
Supplement: S1 Table — (DOCX) [file pone.0205807.s002.docx]

**Table S1.** Clinical characteristics of matched and unmatched metformin users

| Characteristics | Un-matched  (n=179) | Matched  (n=88,866) | *p*-value^#^ |
| --- | --- | --- | --- |
| Male | 108 (60.3%) | 48,334 (54.4 %) | 0.111 |
| Age (mean ± SD) | 60.5 ± 13.3 | 55.9 ± 12.9 | <0.001 |
| Type 1 DM | 5 (2.8%) | 1,904 (2.1%) | 0.548 |
| Co-morbidity |  |  |  |
| COPD | 13 (7.3%) | 4,780 (5.4%) | 0.265 |
| Pulmonary cancer | 1 (0.6%) | 118 (0.1%) | 0.213 |
| Extra-pulmonary cancer | 18 (10.1%) | 2,420 (2.7%) | <0.001 |
| Bronchiectasis | 2 (1.1%) | 731 (0.8%) | 0.661 |
| Psoriasis | 6 (3.4%) | 593 (0.7%) | <0.001 |
| Rheumatoid arthritis | 0 (0%) | 322 (0.4%) | >0.999 |
| Ankylosing spondylitis | 1 (0.6%) | 184 (0.2%) | 0.311 |
| Liver cirrhosis | 2 (1.1%) | 160 (0.2%) | 0.043 |
| Severe autoimmune disease | 0 (0%) | 128 (0.1%) | >0.999 |
| Pneumoconiosis | 0 (0%) | 96 (0.1%) | >0.999 |
| HIV/AIDS | 0 (0%) | 40 (0.05%) | >0.999 |
| Transplantation | 0 (0%) | 40 (0.05%) | >0.999 |
| DM chronic complication* | 12 (6.7%) | 6,062 (6.8%) | >0.999 |
| Low income | 12 (6.7%) | 6,138 (6.9%) | 0.915 |
| Medications |  |  |  |
| Insulin | 13 (7.3%) | 4,740 (5.3%) | 0.251 |
| OHAs other than metformin^$^ | 96 (53.6%) | 58,895 (66.3%) | <0.001 |
| Statins | 45 (25.1%) | 17,773 (20.0%) | 0.086 |
| Aspirin | 38 (21.2%) | 17,634 (19.8%) | 0.642 |
| NSAIDs | 16 (8.9%) | 6,767 (7.6%) | 0.505 |
| CCBs | 62 (34.6%) | 26,315 (29.6%) | 0.141 |
| Corticosteroids | 19 (10.6%) | 4,562 (5.1%) | 0.001 |
| Immunosuppressants & biologicals | 0 (0%) | 9 (0.01%) | >0.999 |
| DMARDs | 0 (0%) | 7 (0.01%) | >0.999 |

Abbreviations: AIDS, acquired immunodeficiency syndrome; CCBs, calcium channel blockers; COPD, chronic obstructive pulmonary disease; DM, diabetes mellitus; DMARDs, disease-modifying antirheumatic drugs; NSAIDs, non-steroidal anti-inflammatory drugs; OHAs, oral hypoglycemic agents; TB, tuberculosis.

Data are expressed as the number (%) unless otherwise mentioned.

^#^ *p* value was calculated by using the *chi*-square test (or Fisher exact test) for categorical variables and *t* test for continuous variables.

* Including diabetic nephropathy, diabetic retinopathy, diabetic neuropathy, and diabetic vasculopathy.

^$^ Including sulfonylurea, meglitinide, alpha-glucosidase inhibitor, thiazolidinedione, dipeptidyl peptidase-4 (DDP4)-inhibitor.
