# Supplementary material for: Metformin use is associated with a low risk of tuberculosis among newly diagnosed diabetes mellitus patients with normal renal function: A nationwide cohort study with validated diagnostic criteria
Source: PLoS One. 2018 Oct 18;13(10):e0205807. doi: 10.1371/journal.pone.0205807 (PMC6193668; doi:10.1371/journal.pone.0205807)
Supplement: S2 Table — (DOCX) [file pone.0205807.s003.docx]

**Table S2.** Results of validation for the diagnostic criteria of tuberculosis (TB)

| Number of patients | | TB suspects in NTUH from 2011 to 2012 | |
| --- | --- | --- | --- |
|  |  | TB | Not TB |
| Fulfilling TB diagnostic criteria used in current study | Yes | 798 | 20 |
|  | No | 7 | 19,895 |

Abbreviations: NTUH, National Taiwan University Hospital;
