# Supplementary material for: Metformin use is associated with a low risk of tuberculosis among newly diagnosed diabetes mellitus patients with normal renal function: A nationwide cohort study with validated diagnostic criteria
Source: PLoS One. 2018 Oct 18;13(10):e0205807. doi: 10.1371/journal.pone.0205807 (PMC6193668; doi:10.1371/journal.pone.0205807)
Supplement: S3 Table — (DOCX) [file pone.0205807.s004.docx]

**Table S3.** Clinical characteristics of metformin users and nonusers among diabetic patients with normal renal function after propensity score matching

| Characteristics | Nonusers  (n=87,592) | Users  (n=87,592) | *p*-value^#^ |
| --- | --- | --- | --- |
| Male | 47,530 (54.3%) | 47,610 (54.4 %) | 0.704 |
| Age (mean ± SD) | 55.9 ± 13.1 | 55.9 ± 12.9 | 0.714 |
| Type 1 DM | 1,852 (2.1%) | 1,846 (2.1%) | 0.934 |
| Co-morbidity |  |  |  |
| COPD | 4,726 (5.4%) | 4,692 (5.4%) | 0.721 |
| Pulmonary cancer | 126 (0.1%) | 117 (0.1%) | 0.523 |
| Extra-pulmonary cancer | 2,414 (2.8%) | 2,393 (2.7%) | 0.770 |
| Bronchiectasis | 707 (0.8%) | 719 (0.8%) | 0.770 |
| Psoriasis | 596 (0.7%) | 587 (0.7%) | 0.816 |
| Rheumatoid arthritis | 329 (0.4%) | 313 (0.4%) | 0.553 |
| Ankylosing spondylitis | 185 (0.2%) | 181 (0.2%) | 0.875 |
| Liver cirrhosis | 161 (0.2%) | 159 (0.2%) | 0.954 |
| Severe autoimmune disease | 128 (0.1%) | 117 (0.1%) | 0.618 |
| Pneumoconiosis | 101 (0.1%) | 92 (0.1%) | 0.563 |
| HIV/AIDS | 49 (0.1%) | 40 (0.05%) | 0.397 |
| Transplantation | 42 (0.05%) | 38 (0.04%) | 0.724 |
| DM chronic complication* | 5,045 (5.8%) | 5,174 (5.9%) | 0.189 |
| Low income | 6,081 (6.9%) | 6,038 (6.9%) | 0.692 |
| Medications |  |  |  |
| Insulin | 4,504 (5.1%) | 4,628 (5.3%) | 0.184 |
| OHAs other than metformin^$^ | 58,934 (67.3%) | 57,994 (66.2%) | <0.001 |
| Statins | 16,729 (19.1%) | 17,483 (20.0%) | <0.001 |
| Aspirin | 17,176 (19.6%) | 17,207 (19.6%) | 0.846 |
| NSAIDs | 6,655 (7.6%) | 6,677 (7.6%) | 0.850 |
| CCBs | 25,948 (29.6%) | 25,751 (29.4%) | 0.287 |
| Corticosteroids | 4,412 (5.0%) | 4,505 (5.1%) | 0.314 |
| Immunosuppressants & biologicals | 9 (0.01%) | 8 (0.01%) | >0.999 |
| DMARDs | 4 (0.005%) | 2 (0.002%) | 0.688 |

Abbreviations: AIDS, acquired immunodeficiency syndrome; CCBs, calcium channel blockers; COPD, chronic obstructive pulmonary disease; DM, diabetes mellitus; DMARDs, disease-modifying antirheumatic drugs; NSAIDs, non-steroidal anti-inflammatory drugs; OHAs, oral hypoglycemic agents; TB, tuberculosis.

Data are expressed as the number (%) unless otherwise mentioned.

^#^ *p* value was calculated by using the *chi*-square test (or Fisher exact test) for categorical variables and *t* test for continuous variables.

* Including diabetic nephropathy, diabetic retinopathy, diabetic neuropathy, and diabetic vasculopathy.

^$^ Including sulfonylurea, meglitinide, alpha-glucosidase inhibitor, thiazolidinedione, dipeptidyl peptidase-4 (DDP4)-inhibitor.
