# Supplementary material for: Metformin use is associated with a low risk of tuberculosis among newly diagnosed diabetes mellitus patients with normal renal function: A nationwide cohort study with validated diagnostic criteria
Source: PLoS One. 2018 Oct 18;13(10):e0205807. doi: 10.1371/journal.pone.0205807 (PMC6193668; doi:10.1371/journal.pone.0205807)
Supplement: S4 Table — (DOCX) [file pone.0205807.s005.docx]

**Table S4.** Clinical characteristics of metformin users with normal renal function stratified by cumulative metformin dose using a cutoff of 150 defined daily doses (cDDDs)

| Characteristics | Metformin cDDDs 90-150  (n=36,756) | Metformin cDDDs > 150  (n=52,110) | *p*-value^#^ |
| --- | --- | --- | --- |
| Male | 19,593 (54.1%) | 28,017 (54.6 %) | 0.163 |
| Age (mean ± SD) | 56.3 ± 13.0 | 55.6 ± 12.8 | <0.001 |
| Type 1 DM | 717 (2.0%) | 1,129 (2.2%) | 0.026 |
| Co-morbidity |  |  |  |
| COPD | 2,072 (5.7%) | 2,620 (5.1%) | <0.001 |
| Pulmonary cancer | 47 (0.1%) | 70 (0.1%) | 0.793 |
| Extra-pulmonary cancer | 974 (2.7%) | 1,419 (2.8%) | 0.504 |
| Bronchiectasis | 295 (0.8%) | 424 (0.8%) | 0.854 |
| Psoriasis | 227 (0.6%) | 360 (0.7%) | 0.184 |
| Rheumatoid arthritis | 160 (0.4%) | 152 (0.3%) | <0.001 |
| Ankylosing spondylitis | 82 (0.2%) | 99 (0.2%) | 0.282 |
| Liver cirrhosis | 67 (0.2%) | 92 (0.2%) | 0.843 |
| Severe autoimmune disease | 54 (0.1%) | 70 (0.1%) | 0.677 |
| Pneumoconiosis | 38 (0.1%) | 54 (0.1%) | 0.990 |
| HIV/AIDS | 19 (0.05%) | 21 (0.04%) | 0.431 |
| Transplantation | 11 (0.03%) | 27 (0.05%) | 0.120 |
| DM chronic complication* | 1,949 (5.4%) | 3,225 (6.3%) | <0.001 |
| Low income | 2,520 (7.0%) | 3,518 (6.8%) | 0.545 |
| Medications |  |  |  |
| Insulin | 1,955 (5.4%) | 2,673 (5.2%) | 0.213 |
| OHAs other than metformin^$^ | 22,210 (61.3%) | 35,784 (69.7%) | <0.001 |
| Statins | 6,295 (17.4%) | 11,188 (21.8%) | <0.001 |
| Aspirin | 6,585 (18.2%) | 10,622 (20.7%) | <0.001 |
| NSAIDs | 2,836 (7.8%) | 3,841 (7.5%) | 0.056 |
| CCBs | 10,125 (27.9%) | 15,626 (30.4%) | <0.001 |
| Corticosteroids | 1,979 (5.5%) | 2,526 (4.9%) | <0.001 |
| Immunosuppressants & biologicals | 2 (0.01%) | 6 (0.01%) | 0.483 |
| DMARDs | 1 (0.002%) | 1 (0.002%) | >0.999 |

Abbreviations: AIDS, acquired immunodeficiency syndrome; CCBs, calcium channel blockers; COPD, chronic obstructive pulmonary disease; DM, diabetes mellitus; DMARDs, disease-modifying antirheumatic drugs; NSAIDs, non-steroidal anti-inflammatory drugs; OHAs, oral hypoglycemic agents; TB, tuberculosis.

Data are expressed as the number (%) unless otherwise mentioned.

^#^ *p* value was calculated by using the *chi*-square test (or Fisher exact test) for categorical variables and *t* test for continuous variables.

* Including diabetic nephropathy, diabetic retinopathy, diabetic neuropathy, and diabetic vasculopathy.

^$^ Including sulfonylurea, meglitinide, alpha-glucosidase inhibitor, thiazolidinedione, dipeptidyl peptidase-4 (DDP4)-inhibitor.
