# Supplementary material for: Metformin use is associated with a low risk of tuberculosis among newly diagnosed diabetes mellitus patients with normal renal function: A nationwide cohort study with validated diagnostic criteria
Source: PLoS One. 2018 Oct 18;13(10):e0205807. doi: 10.1371/journal.pone.0205807 (PMC6193668; doi:10.1371/journal.pone.0205807)

**Figure.** Cumulative defined daily dose (DDD) of metformin in metformin users and nonusers in each year among all enrolled patients with diabetes mellitus (Figure A) and those with normal renal function (Figure B)


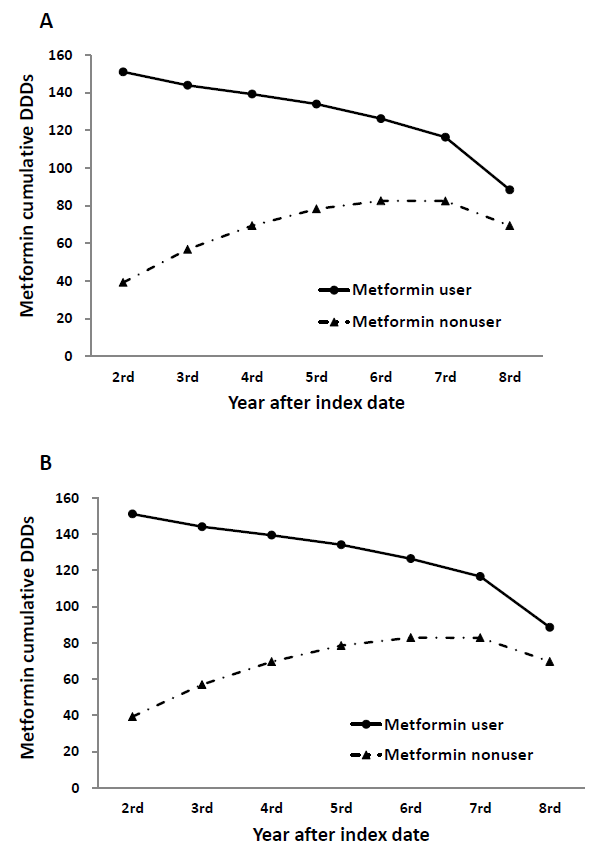

Supplement: S1 File — Cumulative defined daily dose (DDD) of metformin in metformin users and nonusers in each year among all enrolled patients with diabetes mellitus (Figure A) and those with normal renal function (Figure B). (DOCX) [file pone.0205807.s006.docx]
